# DNA repair gene *XRCC1 *polymorphisms and bladder cancer risk

**DOI:** 10.1186/1471-2156-8-13

**Published:** 2007-04-10

**Authors:** Sei Chung Sak, Jennifer H Barrett, Alan B Paul, D Timothy Bishop, Anne E Kiltie

**Affiliations:** 1 Molecular Radiobiology Group, Section of Oncology, Leeds Institute of Molecular Medicine, St James's University Hospital, Leeds, LS9 7TF, UK; 2 Section of Epidemiology and Biostatistics, Leeds Institute of Molecular Medicine, St James's University Hospital, Leeds, LS9 7TF, UK; 3 Department of Urology, St James's University Hospital, Leeds, LS9 7TF, UK

## Abstract

**Background:**

Cigarette smoking and chemical occupational exposure are the main known risk factors for bladder transitional cell carcinoma (TCC). Oxidative DNA damage induced by carcinogens present in these exposures requires accurate base excision repair (BER). The XRCC1 protein plays a crucial role in BER by acting as a scaffold for other BER enzymes. Variants in the XRCC1 gene might alter protein structure or function or create alternatively spliced proteins which may influence BER efficiency and hence affect individual susceptibility to bladder cancer. Recent epidemiological studies have shown inconsistent associations between these polymorphisms and bladder cancer. To clarify the situation, we conducted a comprehensive analysis of 14 XRCC1 polymorphisms in a case-control study involving more than 1100 subjects.

**Results:**

We found no evidence of an association between any of the 14 XRCC1 polymorphisms and bladder cancer risk. However, we found carriage of the variant Arg280His allele to be marginally associated with increased bladder cancer risk compared to the wild-type genotype (adjusted odds ratio [95% confidence interval], 1.50 [0.98–2.28], p = 0.06). The association was stronger for current smokers such that individuals carrying the variant 280His allele had a two to three-fold increased risk of bladder cancer compared to those carrying the wildtype genotype (p = 0.09). However, the evidence for gene-environment interaction was not statistically significant (p = 0.45).

**Conclusion:**

We provide no evidence of an association between polymorphisms in XRCC1 and bladder cancer risk, although our study had only limited power to detect the association for low frequency variants, such as Arg280His.

## Background

Bladder cancer is the fourth most common malignancy in Europeans [1]. Tobacco smoking and occupational exposure to chemicals are two well established risk factors [2,3]. Associated carcinogens damage DNA, and failure of accurate repair can result in mutations which may trigger carcinogenesis.

Interindividual variability in DNA repair capability (DRC) is an important factor influencing an individual's cancer risk [4]. DNA repair gene polymorphisms may contribute to this variation [5]. XRCC1 is an essential DNA repair gene involved in base excision repair (BER) [6]. Spontaneous chromosome aberrations and deletions are seen in XRCC1 mutant cells (EM9), and XRCC1 knock out (-/-) mice are embryonic lethal [7,8].

The XRCC1 gene exhibits polymorphic variations, including three common single nucleotide polymorphisms (SNPs) that result in amino acid substitutions in exon 7 (Arg194Trp), exon 9 (Arg280His) and exon 10 (Arg399Gln). These nonconservative amino acid alterations may influence DRC by altering the protein-protein interactions between XRCC1 and other BER proteins. The Arg399Gln variant was found to be associated with several phenotypic alterations, including higher levels of sister chromatid exchange [9], aflatoxin B1-DNA adducts, glycophorin A mutations [10] and polyphenol DNA adducts [11], although other data found no adverse effect on DRC [12]. One study suggested the 194Trp variant has increased DRC [10]. Functional studies showed that the Arg280His variant has reduced cellular BER efficiency [13,14].

Epidemiological studies have shown significant associations between the Arg399Gln variant and various cancers (reviewed by Goode et al. [15]), but in bladder cancer the results have been inconsistent [16-18]. A recent meta-analysis of 38 case-control studies by Hu et al. [19] concluded that the Arg194Trp variant had a protective effect on cancer risk, while individuals carrying the Arg280His variant allele had increased cancer risk compared to those with the wildtype genotypes (odds ratio [95% confidence intervals], 1.19 [1.00–1.42]).

In addition to coding SNPs, non-coding XRCC1 polymorphisms may also affect DRC by altering the splice site or transcription efficiency. Recently, Hao et al. [20] discovered a novel T-77C polymorphism (rs3213245) in the XRCC1 gene which contributes to diminished promoter activity and increased risk of non-small cell lung cancer.

In the present study, we performed a comprehensive analysis of 14 potentially functional polymorphisms (coding and non coding) in XRCC1 to investigate their associations with bladder cancer. Furthermore, we constructed XRCC1 haplotypes and assessed interactions with smoking and occupational exposure.

## Results

### Subject characteristics

Demographic details for each subject have been described previously [21]. The majority of subjects were Caucasian (98.6%) with no difference in mean age (cases 72.8 years; controls 71.9 years). There was no significant difference in mean age of community and hospital controls (p = 0.19) and no difference in smoking or occupational exposure rates. There were however more men in the hospital group (P < 0.001) because of an attempt to obtain a similar overall sex ratio to the cases. Cases were more likely than controls to be smokers (78% vs. 64%, p < 0.001), male (70.9% vs. 65.5%, p = 0.05), have previous occupational exposure (27.4% vs. 16.8%, p < 0.001) and a positive family history of bladder cancer (4.8% vs. 2.2%, p = 0.02).

### Genotyping

The genotyping success rate was 95.0% (range 90.1 to 98.1%). The control genotype distributions were all in Hardy-Weinberg equilibrium. Hospital and community control genotypes only differed in one polymorphism, Gln632Gln at the 5% level (minor allele frequency 0.48 vs.0.41, p = 0.04), consistent with random variation given the number of polymorphisms examined so the two control groups were combined to increase the study power.

We found no variant allele for Val72. The remaining polymorphisms exhibited strong linkage disequilibrium (LD) (Table 1). The crude and adjusted odds ratio for all 14 XRCC1 polymorphisms and bladder cancer risk are shown in Table 2. No polymorphism showed association with bladder cancer risk at the 5% significance level, although individuals carrying the 280His variant allele had a marginally significant increased risk of bladder TCC compared to those carrying the homozygote wildtype genotype (adjusted OR [95% CI], 1.50 [0.98–2.28], p = 0.06).

**Table 1:** The linkage disequilibrium (LD) between all 14 XRCC1 polymorphisms

No	Polymorphisms	**1**	**2**	3	4	**5**	6	**7**	**8**	9	**10**	**11**	**12**	**13**	**14**
1	EX1-1139 D' (r2)	***0.20 (0.13)***													
2	EX1-1128	0.82 (0.57)	***0.22 (0.13*)**												
3	EX1-900	0.92 (0.30)	0.98 (0.39)	***0.41 (0.22)***											
4	EX-128	0.89 (0.27)	0.95 (0.35)	**1 (0.93)**	***0.42 (0.22)***										
5	EX-52	1 (0.01)	1 (0.01)	1 (0.02)	0.68 (0.02)	***0.03 (0.02)***									
6	Val72Ala	N/A	N/A	N/A	N/A	N/A	***0 (0.03)***								
**7**	Arg194Trp	1 (0.02)	1 (0.02)	0.97 (0.04)	0.97 (0.04)	1 (0)	N/A	***0.06 (0.12)***							
8	IVS7-33	0.04 (0)	0.05 (0)	0.06 (0)	0.06 (0)	0.15 (0)	N/A	0.13 (0)	***0.46 (0.11)***						
9	Pro206Pro	0.89 (0.24)	0.96 (0.32)	**1 (0.83)**	**0.97 (0.83)**	0.39 (0.01)	N/A	0.98 (0.05)	0.06 (0)	***0.44 (0.26)***					
10	Arg280His	0.65 (0)	0.93 (0.01)	0.79 (0.02)	0.74 (0.02)	1 (0)	N/A	1 (0)	1 (0.04)	0.70 (0.02)	***0.04 (0.10)***				
11	Arg399Gln	0.87 (0.11)	0.93 (0.14)	1 (0.39)	0.96 (0.39)	0.04 (0)	N/A	1 (0.04)	0.08 (0)	0.99 (0.47)	1 (0.03)	***0.37 (0.23)***			
12	Gln632Gln	0.86 (0.24)	0.95 (0.32)	**0.99 (0.84)**	0.91 (0.77)	1 (0.02)	N/A	0.91 (0.04)	0.06 (0)	**0.97 (0.90)**	0.61 (0.01)	1 (0.45)	***0.56 (0.29)***		
13	EX17-123	0.69 (0.01)	0.76 (0.01)	0.86 (0.03)	0.92 (0.03)	1 (0)	N/A	1 (0)	0 (0)	0.94 (0.04)	0.82 (0)	0.98 (0.03)	0.88 (0.04)	***0.06 (0.13****)*	
14	EX17-127	0.69 (0.03)	0.75 (0.04)	0.95 (0.33)	0.96 (0.32)	1 (0.01)	N/A	0.77 (0.01)	0.08 (0)	1 (0.30)	1 (0.01)	1 (0.16)	0.99 (0.31)	0.11 (0)	***0.21 (0.09)***

Values in bold italics represent the observed followed by estimated (in round bracket) control minor allele frequencies from public databases.

* LD is presented in pairwise D' followed by r^2 ^in round bracket.

N/A = Not available because of no pairwise variant genotypes in rare polymorphisms.

Values in bold are polymorphisms in high LD (D' ≥ 0.90and r2 ≥ 0.80).

**Table 2:** The association between the 14 XRCC1 polymorphisms and bladder cancer risk

Polymorphisms*	**Genotypes**	**Controls, n (%)**	**Cases, n (%)**	**aOR (95% CI)**	**P value**
EX1-1139	C/C	328 (61.5)	295 (59.0)	1.00	reference
rs2682586	C/T	187 (35.1)	181 (36.2)	1.07 (0.82–1.39)	0.62
Promoter	T/T	18 (3.4)	24 (4.8)	1.42 (0.74–2.71)	0.29
					
	T allele freq.	0.209	0.229		

EX1-1128 rs2682585	C/C C/T	319 (60.3) 189 (35.7)	287 (59.1) 177 (36.4)	1.00 1.05 (0.81–1.37)	reference 0.71
Promoter	T/T	21 (4.0)	22 (4.5)	1.15 (0.61–2.18)	0.66
					
	T allele freq.	0.218	0.227		

EX1-900	-/-	196 (35.5)	173 (34.0)	1.00	reference
rs3213239	-/+	267 (48.2)	256 (50.3)	1.04 (0.79–1.36)	0.79
Promoter	+/+	91 (16.4)	80 (15.7)	1.02 (0.71–1.48)	0.91
					
	+ allele freq.	0.405	0.409		

EX-128	C/C	187 (33.6)	174 (32.8)	1.00	reference
rs3213245	C/T	275 (49.4)	266 (50.2)	1.00 (0.76–1.31)	0.97
5'UTR	T/T	94 (16.9)	90 (17.0)	1.06 (0.74–1.52)	0.76
					
	T allele freq.	0.416	0.421		

EX-52	C/C	548 (97.2)	520 (97.6)	1.00	reference
rs2307187	C/T	16 (2.8)	13 (2.4)	0.91 (0.43–1.92)	0.80
5'UTR	T/T	0	0	NC	NC
					
	T allele freq.	0.014	0.012		

Val72Ala	C/C	562 (100)	521 (100)	1.00	reference
rs25496	C/T	0	0	NC	NC
Exon 3	T/T	0	0	NC	NC
					
	T allele freq.	0	0		

Arg194Trp	C/C	498 (88.6)	476 (89.0)	1.00	reference
rs1799782	C/T	61 (10.9)	56 (10.4)	0.95 (0.64–1.41)	0.81
Exon 6	T/T	3 (0.5)	3 (0.6)	1.01 (0.19–5.23)	0.99
	C/T + T/T	64 (11.4)	59 (11.0)	0.95 (0.65–1.40)	0.81
					
	T allele freq.	0.060	0.058		

IVS7-33	C/C	518 (91.7)	484 (89.6)	1.00	reference
rs1799780	C/T	47 (8.3)	55 (10.1)	1.30 (0.85–1.97)	0.22
Intron 7	T/T	0 (0)	1 (0.2)	∞	NC
	C/T + T/T	47 (8.3)	56 (10.4)	1.33 (0.88–2.01)	0.18
					
	T allele freq.	0.042	0.053		

Pro206Pro	G/G	170 (31.2)	162 (31.4)	1.00	reference
rs915927	G/A	270 (49.5)	260 (50.5)	0.96 (0.73–1.28)	0.80
Exon 7	A/A	105 (19.3)	93 (18.1)	0.91 (0.64–1.31)	0.63
					
	A allele freq.	0.440	0.433		

Arg280His	G/G	516 (92.1)	456 (88.9)	1.00	reference
rs25489	G/A	41 (7.3)	54 (10.5)	1.52 (0.98–2.34)	0.06
Exon 9	A/A	3 (0.6)	3 (0.6)	1.25 (0.25–6.34)	0.78
	G/A + A/A	44 (7.9)	57 (11.1)	1.50 (0.98–2.28)	0.06
					
	A allele freq.	0.042	0.058		

Arg399Gln	G/G	226 (40.4)	218 (41.0)	1.00	reference
rs25487	G/A	259 (46.2)	248 (46.6)	0.97 (0.75–1.26)	0.83
Exon 10	A/A	75 (13.4)	66 (12.4)	0.94 (0.64–1.39)	0.76
					
	A allele freq.	0.365	0.357		

Gln632Gln	G/G	176 (31.4)	173 (32.2)	1.00	reference
rs3547	G/A	275 (49.0)	268 (49.9)	0.94 (0.72–1.24)	0.67
Exon 17	A/A	110 (19.6)	96 (17.8)	0.87 (0.61–1.23)	0.43
					
	A allele freq.	0.441	0.428		

EX17-123	-/-	492 (89.5)	448 (88.4)	1.00	reference
rs3213401	-/+	55 (10.0)	56 (11.0)	1.18 (0.79–1.76)	0.43
3'UTR	+/+	3 (0.5)	3 (0.6)	0.78 (0.15–4.09)	0.77
	-/+ plus +/+	58 (10.5)	59 (11.6)	1.15 (0.78–1.70)	0.48
					
	+ allele freq.	0.055	0.061		

EX17-127	G/G	337 (64.1)	320 (65.6)	1.00	
rs2682558	G/A	162 (30.8)	142 (29.1)	0.91 (0.69–1.20)	0.48
3'UTR	A/A	27 (5.1)	26 (5.3)	1.13 (0.64–1.99)	0.68
					
	A allele freq.	0.205	0.199		

* Polymorphisms included name, dbSNP reference number and its location in XRCC1 gene.

aOR = odds ratio adjusted for subject's age, gender, smoking, occupational exposure and family history

-= wildtype without insertion, + = variant with insertion of GGGAATC

### Gene-environment interactions

Individuals were stratified by smoking status (non-smokers, and ex- and current smokers) and occupational exposure (exposure and no exposure). Current smokers carrying the 280His variant allele were associated with a non-significant two to three-fold increased bladder TCC risk compared to those carrying the homozygous wildtype genotype (adjusted OR [95%CI], 2.52 [0.87–7.31], p = 0.09) (Table 3). We found no evidence of gene-environment interactions between Arg194Trp, Arg280His or Arg399Gln and tobacco smoking (p-values for departure from multiplicative joint effect = 0.32, 0.45 and 0.25 respectively). Similarly, there was no evidence of interaction between occupational status and any of the three non-synonymous polymorphisms (p-values for departure from multiplicative joint effect = 0.33, 0.40 and 0.12 respectively).

**Table 3:** Stratified analysis by smoking and occupational status for XRCC1 Arg280His polymorphism

**Exposure**	**status**	**Genotypes**	**Control (n)**	**Cases (n)**	**Crude OR (95%CI)**	**Adjusted OR (95%CI)**	**P value**
Smoking	Non-smoker	G/G	174	103	1.00	1.00	
		G/A + A/A	16	12	1.27 (0.58–2.78)	1.13 (0.50–2.56)	0.77
	Ex smoker	G/G	253	244	1.00	1.00	
		G/A + A/A	23	29	1.31 (0.74–2.32)	1.30 (0.73–2.33)	0.38
	Current smoker	G/G	89	108	1.00	1.00	
		G/A + A/A	5	16	2.64 (0.93–7.48)	2.52 (0.87–7.31)	0.09

Occupation	No occupational exp	G/G	429	327	1.00	1.00	
		G/A + A/A	35	43	1.61 (1.01–2.58)	1.55 (0.96–2.51)	0.07
	Occupational exp	G/G	87	129	1.00	1.00	
		G/A + A/A	9	14	1.05 (0.43–2.53)	1.03 (0.42–2.54)	0.95

* Adjusted OR for subject's age, gender, smoking, occupational exposure and family history

### Haplotype analysis

We selected the three coding polymorphisms with amino acid substitutions previously commonly investigated, namely Arg194Trp, Arg280His and Arg399Gln to construct XRCC1 haplotypes. Four common haplotypes were estimated to account for over 99% of all haplotypes (Table 4). There was no significant difference in haplotype frequency between cases and controls (p = 0.60).

**Table 4:** Estimated XRCC1 haplotype frequencies in cases and controls

**Arg194Trp**	**Arg280His**	**Arg399Gln**	**Controls haplotype (%)**	**Cases haplotype (%)**
C	G	G	52.9	52.6
C	G	A	36.9	36.1
C	A	G	4.2	5.5
T	G	G	6.0	5.8

Total			100.0	100.0

## Discussion

To date, this is the most comprehensive investigation on the association between XRCC1 polymorphisms and bladder TCC risk. We chose to study one gene comprehensively rather than studying a small number of SNPs in multiple genes, as others have done, for a number of reasons. Firstly, non-coding SNPs in the promoter region, 3' or 5'UTR or putative splice sites have been shown to influence DNA repair capacity [20]. Secondly this approach allows for construction and analysis of haplotypes and provides data on linkage disequilibrium between SNPs in the gene. We found none of the 14 XRCC1 polymorphisms was associated with bladder cancer risk. Our results on the Arg399Gln were consistent with other studies in bladder cancer [17,18]. Although our study was larger and had sufficient power, our results did not support the finding of Kelsey et al. [16] that the variant 399Gln was protective against bladder cancer. We also found no association between Arg194Trp and bladder cancer risk which is consistent with Wu et al. [18].

Recently, Hao et al. [20] in 1024 Chinese lung cancer patients and 1118 controls, showed that a 5'UTR SNP (T-77C, rs3213245), is associated with an increased risk of lung cancer, with OR for carriage of the variant allele of 1.46 (95% CI 1.18–1.82) compared to wildtype genotype. In functional studies the C allele-containing promoter had reduced transcriptional activity. We, however, showed no association for this SNP and increased bladder cancer risk.

Our data provided borderline evidence of association between Arg280His and bladder TCC with an estimated 50% increase in risk to individuals carrying the 280His variant (adjusted OR [95%CI], 1.50 [0.98–2.28]) but this study only had limited power (55%) to detect association with this variant. The association between XRCC1 Arg280His and an increased risk of bladder cancer is biologically plausible [13,14]. Takanami et al. [14] showed that the XRCC1 Arg280His variant protein is defective in its efficient localization to a damaged DNA site and thus impaired the cellular BER efficiency. Furthermore, the recent meta-analysis by Hu et al. [19] also found the 280His variant to be associated with increased cancer risk. However, the only previous bladder cancer study by Stern et al. [22] (235 cases and 213 controls) found no association. Our discrepant findings may be explained by false positive or false negative results, or the different populations studied.

This study was potentially limited by selection bias since both community and hospital controls were included. Although there were differences in the genotype distributions of one polymorphism significant at the 5% level, we believe it unlikely that use of these two control groups influenced the results. Recall bias for smoking, occupational exposure and family history cannot be excluded. Our results must be interpreted cautiously bearing in mind multiple testing as 14 XRCC1 polymorphisms were selected. However, these tests are highly correlated since numbers of polymorphisms were in almost perfect LD.

Exposure to tobacco smoke or to the occupational risk factors studied causes damage to DNA, and these exposures have been shown to increase the risk of bladder cancer. Exposure to these chemicals in an individual with an inefficient DNA repair mechanism might be expected to elevate risk more substantially. We therefore tested the hypothesis that the joint effect on risk of exposure and genotype was greater than multiplicative (gene-environment interaction). Stratified analysis by smoking and chemical exposure did not show any significant gene environment interaction. Interestingly, the 280His variant allele seemed to have a greater influence on bladder cancer risk particularly among current smokers (adjusted OR [95%CI], 2.51 [0.87–7.31], p = 0.09) but the likelihood ratio test for interaction was not significant (p = 0.45). A few other studies have investigated interactions between XRCC1 polymorphisms and smoking on bladder cancer risk [16,23,24]. None has shown evidence of interaction, although it should be borne in mind that most studies to date, including our own, only have sufficient power to detect strong interactions.

## Conclusion

This study provides no evidence of an association between polymorphisms in XRCC1 and bladder cancer risk. However, although the study is relatively large (547 cases and 579 controls), there is limited power to detect the association for low frequency variants such as Arg280His, and the data are consistent with a modestly increased risk for carriers of the variant allele.

## Methods

### Study population

A detailed description of the study population and design has been given elsewhere [21]. In brief, 547 cases of bladder transitional cell carcinoma (TCC) were recruited from August 2002 to April 2004 at our institution in Leeds, UK, after obtaining ethical approval from the Leeds (East) Local Research Ethical Committee. Cancer-free controls (n = 579) were recruited from the community from 1997 to 2000 (n = 227) as part of a previous colorectal cancer study [25] and from the hospital otolaryngology and ophthalmology departments in 2002 to 2004 (n = 352) in the same region. The participation rate for cases and controls were 99% and 80% respectively.

Each subject donated a blood sample and completed a structured health questionnaire regarding smoking, occupational and family history. Occupational exposure was defined as participating in occupations involving rubber/plastics industries, laboratories, printing, paints, dyes or diesel fumes.

### Genotyping

We selected XRCC1 polymorphisms based on potential function from the public domain of the Environmental Genome Project (EGP) via the National Centre for Biotechnology Information[26]. XRCC1 polymorphisms with allele frequencies of more than 1% located in the promoter region (up to 1000 bases upstream of the gene), 5'and 3' untranslated regions (UTR), exons, and intronic regions with known or potential splicing effects (within 100 bases up and downstream of exons) were selected (Table 1).

Genotyping was performed using the allelic discrimination 5' nuclease assay (Taqman) without prior knowledge of the subject's clinical status, as previously described [21]. Primers and probes are listed in Table 5. As a quality control measure, 5% of the samples (n = 57) were regenotyped with 100% concordance.

**Table 5:** List of primers and probes for 14 *XRCC1 *polymorphisms

Polymorphisms	Forward primers	Reverse primers	VIC probes	FAM probes
rs2682586	GTCCCAGATTGAGAGAGAGAGAGAT	CCCGTTCACCTTGAGGACTTG	CTGAAGGCTCTCTCTCT	TGAAGGCTCTTTCTCT
rs2682585	CTCCCCGTAGGGTGAATGTG	CTGGTCCCCAGCTTTTATAGGAA	CAGACCCGCCCCTC	CAGACCCACCCCTC
rs3213239	CACCACCCTGTTTTCTCACCTT	AGGCCCAACTCCGTCTTG	CAATGGGCCGGCCGT	CAAACAACAATGGGCCGT
rs3213245	CGCGCTTGCGCACTTTAG	GCCAGAAGGATGAGGTAGAGTATG	CCCGCTCCCTCCCA	CCGCCCCCTCCCA
rs2307187	CCCCATACTCTACCTCATCCTTCTG	GCTGCAGGACACGACATG	CCGGCATGTCAACGT	TCCGGCATATCAACGT
rs25496	TGGGAATGATGGCTCAGCTTT	CCTGCTTACCTCATAGTCTTGCT	ACTGCCCACCAGCAC	TGCCCGCCAGCAC
rs1799782	AGGATGAGAGCGCCAACTC	ACTCAGGACCCACGTTGTC	TTGTTGATCCGGCTGAA	TTGTTGATCCAGCTGAA
rs1799780	CCATAGATAGGAGTGAAAGGGTCTTG	GCTGTGACTATGAAGGGAGAAAGTG	CAGGATGAGAGGGCTGA	CAGGATGAGAAGGCTGA
rs915927	TCCACTTTCTCCCTTCATAGTCACA	AGGGTAGCAGCTGCATAGC	CCAGCGACCCAGCAG	CCAGCGACCCGGCAG
rs25489	CCAGTGGTGCTAACCTAATCTACTCT	GCTCGGGCAGGGACTG	CTCCAACTCGTACCCC	TCCAACTCATACCCC
rs25487	GTGGGTGCTGGACTGTCA	GCAGGGTTGGCGTGTGA	CCTCCCGGAGGTAA	CCCTCCCAGAGGTAA
rs3213401	GAGTTGGTTCTCATCCAAGA	AAGATACAGGTGTGGCTCAG	*	*
rs2682558	CCTTATCCCTGTGTTGGCAAGAG	GGCTCAGAGGGCCAGAAAA	CAGATTCCCAGTTCCCT	CAGATTTCCAGTTCCCT
rs3547	TGTGTGTGTGTGTGTGTATAGCA	GCAGAAGTTACTTCCTCACCATCTC	CCGCAGGCCTGAAG	CCGCAAGCCTGAAG

Probes are not required because the variant allele involved deletion of 7 nucleotides which is sufficient to be detected by PCR product size.

### Statistical analysis

All data were analyzed using Stata Statistical Software Release 8 (Stata Corporation, 2003, College Station, Texas). Hardy-Weinberg equilibrium analyses were performed to compare observed and expected genotype frequencies using a chi-squared goodness-of-fit test. The linkage disequilibrium (LD) between polymorphisms was estimated by pairwise Lewontin's D' and r^2 ^using the STATA pwld function [27]. The odds ratio (OR) and 95% confidence interval (CI) for bladder cancer associated with each genotype were calculated using logistic regression analysis, both without adjustment and adjusted for established bladder cancer risk factors (age, sex, cigarette smoking, occupational exposure and family history).

Haplotypes were estimated by the Expectation Maximization (EM) algorithm [28] and their frequencies in cases and controls compared using a likelihood ratio test with a permutation procedure to obtain empirical p-values as implemented in EHPLUS [29]. With a sample size of 547 cases and 579 controls, we had an 90%, 78% and 55% power to detect an OR of 1.50 for carriage of the minor allele of frequency 0.20, 0.10 and 0.05 respectively (2 sided, p = 0.05). Gene-environment interaction was assessed by stratification of subjects based on smoking status and occupational exposure, and p values are calculated based on the likelihood-ratio test, comparing models with and without an interaction term.

## Authors' contributions

SCS recruited the cases and the majority of the controls, co-ordinated the genotyping, performed the main statistical analyses and interpretation of results, drafted the manuscript.

JHB participated in the design of the study, advised on and carried out some of the statistical analyses and interpreted the results, and helped to draft and redraft the manuscript.

ABP gave clinical advice on patients, participated in the design of the study and critically appraised the manuscript.

DTB helped in the conception of the study, the interpretation of the results and redrafting of the manuscript.

AEK conceived the study, facilitated the genotyping and helped to redraft the manuscript.

All authors read and approved the final manuscript.
